# Competences for Providing Oral Health Care to Care‐Dependent Older Adults—Defining Learning Objectives for the German Undergraduate Dental Curriculum Through a Delphi Study

**DOI:** 10.1111/eje.70094

**Published:** 2026-01-16

**Authors:** Anna‐Lena Hillebrecht, Anna Greta Barbe, Susanne Krämer, Marc Auerbacher, Wiebke Semper‐Hogg, Benedikt C. Spies, Philipp Linde, Cornelia Erfurt‐Berge, Anastassia Kossioni, Erik Farin‐Glattacker, Kerstin Bitter

**Affiliations:** ^1^ Department of Prosthetic Dentistry, Faculty of Medicine, Medical Center – University of Freiburg, Center for Dental Medicine University of Freiburg Freiburg Germany; ^2^ Polyclinic for Operative Dentistry and Periodontology, Faculty of Medicine and University Hospital Cologne University of Cologne Cologne Germany; ^3^ Special Care Dentistry Unit, Facultad de Odontologia Universidad de Chile Santiago Chile; ^4^ Department of Conservative Dentistry and Periodontology University Hospital, LMU Munich Munich Germany; ^5^ Department of Oral and Maxillofacial Surgery, Faculty of Medicine, Medical Center – University of Freiburg, Center for Dental Medicine University of Freiburg Freiburg Germany; ^6^ Department of Radiation Oncology, Cyberknife and Radiation Therapy, Faculty of Medicine and University Hospital of Cologne University of Cologne Cologne Germany; ^7^ Department of Dermatology, Uniklinikum Erlangen Friedrich‐Alexander University Erlangen‐Nürnberg Erlangen Germany; ^8^ Department of Prosthodontics, Dental School National and Kapodistrian University of Athens Athens Greece; ^9^ Section of Health Care Research and Rehabilitation Research, Faculty of Medicine, Medical Center – University of Freiburg, Institute of Medical Biometry and Statistics University of Freiburg Freiburg im Breisgau Germany; ^10^ Department for Operative Dentistry and Periodontology, Medical Faculty Martin‐Luther University Halle‐Wittenberg Halle (Saale) Germany

**Keywords:** Delphi study, dependent older people, gerodontology, graduate profile, learning objectives, oral health care

## Abstract

**Background/Aim:**

One of the largest unmet dental care needs in Germany concerns dependent older people (DOP). The aim of this Delphi study was to define learning objectives (LOs) for the dental curriculum, specifying the skills, abilities, and attitudes necessary to enable and motivate future dentists to provide oral health care to DOP.

**Method:**

LOs from international guidelines for special care dentistry and gerodontology were mapped and aligned with the current guidelines of the German dental curriculum. Based on this alignment, comprehensive LOs were formulated and ranked through a Delphi process. The target group consisted of dental education experts from German universities (14 in first round/12 in second round), all of whom had expertise in gerodontology or special care dentistry.

**Results:**

A framework consisting of six competence areas was established: (1) Care dependency in older individuals, (2) Access & barriers to oral health, (3) Legal aspects, (4) Communication, (5) Effects of medical and functional condition on oral health, (6) Clinical management of DOP. The participants agreed on a total of 43 LOs. After refinement, a graduate profile for dental students in Germany with 21 LOs with a focus on the oral health of DOP was developed.

**Discussion:**

The results of this study can be used to provide standardised education about dental care of DOP in Germany. By implementing the LOs and the graduate profile to the undergraduate dental curriculum, students should be prepared for the requirements of dental care for DOP after graduation.

## 1 Introduction

Population ageing, driven by increased longevity and decreased fertility, is affecting many developed countries [1, 2]. With advancing age, the likelihood of requiring care increases, leading to a growing number of dependent older people (DOP) [2].

DOP are older adults for whom regular oral self‐care and/or access to dental care is constrained by physical, cognitive, sensory, communicative, or environmental limitations. This includes individuals meeting at least one of the following: inability to perform daily oral hygiene independently; insufficient cooperation with dental examination or treatment to the usual extent; inability to access a dental practice without substantial assistance due to lack of accessibility; or need for adapted modes of communication beyond usual practice.

In the dental context, care independent older adults (IOA) are characterised by their ability to manage their oral health largely on their own and to regularly utilise dental services. In contrast, DOP rely on external support for daily oral hygiene and for accessing emergency and regular dental care [3]. Their dental care often requires an approach that considers their heterogenous physical and cognitive limitations as well as their care support environment [4]. At the point when individuals become care dependent they often have some natural teeth and/or dental prosthetic restorations such as removable dentures or implant‐supported restorations, which necessitate regular dental check‐ups and additional support with daily oral care to prevent oral diseases [5, 6, 7]. Oral health plays a crucial role in overall health, well‐being, and quality of life, particularly in the presence of age‐related limitations and illnesses [8, 9]. However, DOP currently face restricted access to dental care despite experiencing poorer oral health compared to other population groups [10, 11, 12]. Importantly, this applies regardless of living or care arrangements: barriers to oral health exist both for older adults receiving community‐based support at home and for residents in long‐term care facilities, where organisational structures, staffing, and accessibility constraints may additionally impede dental treatment. In response to the rapidly changing demographic landscape, the implementation of dental interventions appropriate for DOP is crucial to meet the evolving oral healthcare demands [13]. There is a need to ensure mandatory inclusion of sufficient and specific training in gerodontology (GD) to prepare dental graduates for a growing older population with various levels of frailty and care dependency [14, 15, 16]. Teaching content regarding the provision of dental care for DOP has not been widely implemented [17]. In 2017, a European survey showed that GD was taught in 86.2% of the responding dental schools with large variation in curriculum content and educational methods. Almost 30% of respondent schools did not teach medical problems in old age and 36% did not provide clinical teaching in GD [18]. In a field that requires collaboration across multiple disciplines, such as geriatrics, psychiatry, nursing, social care and others, having a comprehensive guideline for teaching is essential to ensure that all relevant aspects are adequately addressed. Until now, German universities were completely free to decide whether they will teach GD or not [19]. This is currently changing due to the new German licencing regulations that include interdisciplinary lectures in GD [20]. A further important opportunity for GD education arises from the recent amendment to the European Commission regulation, which now mandates GD training for dental students across Europe [21]. The dental curriculum should equip students with the essential knowledge, skills, and attitudes to address the oral health needs of IOA and DOP within their communities [22]. GD is a crucial aspect of dental education, emphasising the importance of evaluating both the competencies of graduates and the existing curriculum [23]. Moreover, clinical GD education must ensure adequate and high‐quality experiences in treating older people with various levels of care dependency and in various locations [24]. A significant challenge in GD education lies not solely in the transmission of knowledge and skills but also in effectively fostering students' attitudes and motivation [25, 26]. Learning objectives (LOs) are essential to guide and focus dental students' learning, ensure effective assessment, maintain educational consistency, develop necessary skills and design a coherent curriculum. These frameworks not only standardise learning content but also foster consensus among institutions and educators, facilitating a structured and individualised approach to competency acquisition. Defining specific LOs ensures standardised training, enabling students to provide optimal care for DOP in Germany. A Delphi approach can be used to define content and LOs of required changes in a healthcare curriculum [27, 28, 29, 30]. The aim of this study was to obtain a consensus on the LOs that specify the competencies of German dental graduates in dental care provision for DOP based on relevant international curriculum guidelines and experts' opinions in GD and dental education in Germany.

## 2 Methods

LOs of the international guidelines for special care dentistry (SCD) [31] and GD [24] were aligned with the standard LOs for undergraduate dental curriculum in Germany (‘impp‐Gegenstandskatalog Zahnmedizin’, Catalogue of Examination Subjects, CES). These two guidelines [24, 31] were selected because both focus on the competencies required to provide dental care for DOP. The CES defines the examination‐relevant content for dental education in Germany, ensuring a standardised and practice‐oriented curriculum that aligns with scientific advancements and modern patient care requirements. In order to analyse alignment between the guidelines and the German curriculum, the study team mapped the contained LOs. According to the matching LOs, 41 LOs were formulated and assigned to a framework based on six domains/competence areas: (1) General health of care dependency in older individuals, (2) Access & barriers to oral health, (3) Legal aspects, (4) Communication, (5) Effects of medical and functional condition on oral health, (6) Clinical management of DOP (Supporting Information).

### 2.1 Delphi Survey

To refine the framework, an online Delphi survey was conducted between July 2024 and August 2024. Experts who specialise in GD, publish scientifically in this field, and teach dental students at a German university were approached. The target sample (14 in first round/12 in second round) were dentists who are experts in dental education and gerodontology or special care dentistry in Germany.

### 2.2 Delphi Structure

The data were collected via Google Forms, an online survey service. The quantitative data were analysed by calculating descriptive statistics, and the qualitative data were analysed by identifying common themes through inductive coding. The framework consisting of LOs and competence areas was delivered to the Delphi panel. Each learning goal was assessed for its relevance using a five‐point scale, for example, 5 = not relevant at all, 4 = not very relevant, 3 = quite relevant, 2 = relevant, 1 = very relevant. Additionally, the panellists were invited to provide feedback on the clarity, relevance, and importance of each learning objective.

Very high agreement was defined as a median of 1, an interquartile range (IQR) of 0 and ≥ 80% scoring a 1 or 2. An IQR of 0 indicates that there is no variability within the middle 50% of the data, implying that the first and third quartiles are identical. Furthermore, high agreement was median 1, IQR ≤ 1 and ≥ 80% scoring a 1 or 2. We feedback recommendations with moderate and low agreement only. Moderate agreement or consensus was defined as a median of 1–2, IQR ≤ 2 and ≥ 60% scoring a 1 or 2. Low agreement (no consensus) was a median of 1–2, and (IQR ≤ 2 or IQR ≥ 60% scoring a 1 or 2). Those with medians between 2 and 3 as showing no agreement were rejected. In parallel, we defined, for example, very high disagreement with median 5 and IQR = 0 and ≥ 80% scoring 4 or 5 (full consensus on not relevant) [32, 33]. Between Round 1 and Round 2, all qualitative comments provided by the expert panel were extracted and thematically coded. Feedback primarily addressed wording ambiguities in learning outcomes, overlapping competencies, and insufficient specificity in operational verbs. All suggested modifications were discussed by the core research team.

### 2.3 Second Delphi Round

**FIGURE 1:**
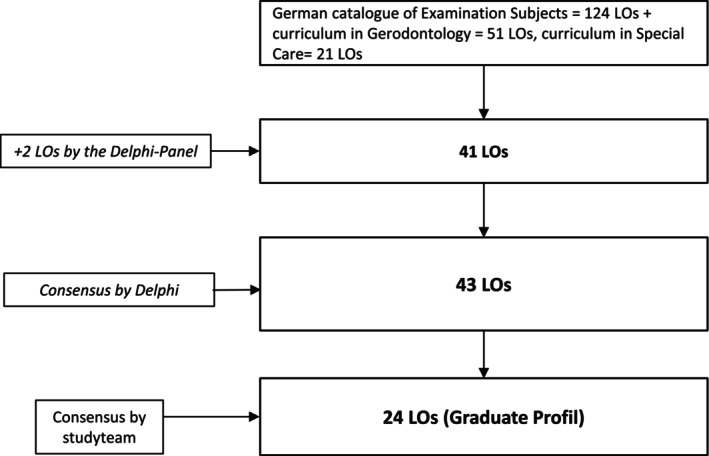
Flowchart for the process of defining learning objectives (LOs) and graduate profile.

In concordance with the Delphi methodology, LOs for which there was a high level of agreement and consensus were removed. LOs for which there were substantial comments or wording suggestions were reworded and included in round 2 for further voting. The second round contained 11 LOs, 9 LOs from the first round and two new LOs, which were added corresponding to the experts' feedback. The study team (G.B., A.K, K.B., A.L.H) refined and streamlined the initial 41 LOs inclusive of the two new LOs, incorporating expert feedback from the Delphi process. This iterative approach has been utilised to guarantee that the newly formulated LOs encompass all the critical skills and knowledge necessary to facilitate dentistry for the DOP, while ensuring that the curriculum does not become excessively burdensome. The refinement process was concluded with the formulation of 24 learning objectives (Graduate Profile). (Figure 1).

### 2.4 Developer and Governance Groups

A.L.H. collected data for the competency framework development and collaborated with A.K., G.B., S.K., P.L., W.S.H. and M.A. to create the framework. K.B., F.G. oversaw the project and provided methodical feedback. Regular online meetings were held to review the data and its implications for successive framework drafts. The developers and governance groups had expertise in GD (A.K., M.A., G.B., A.L.H.), SCD (S.K., M.A., A.L.H.), medical education (P.L., C.E.B.), and dental education (A.K., W.S.H., K.B., G.B., M.A., S.K., B.S., A.L.H.).

## 3 Results

**FIGURE 2:**
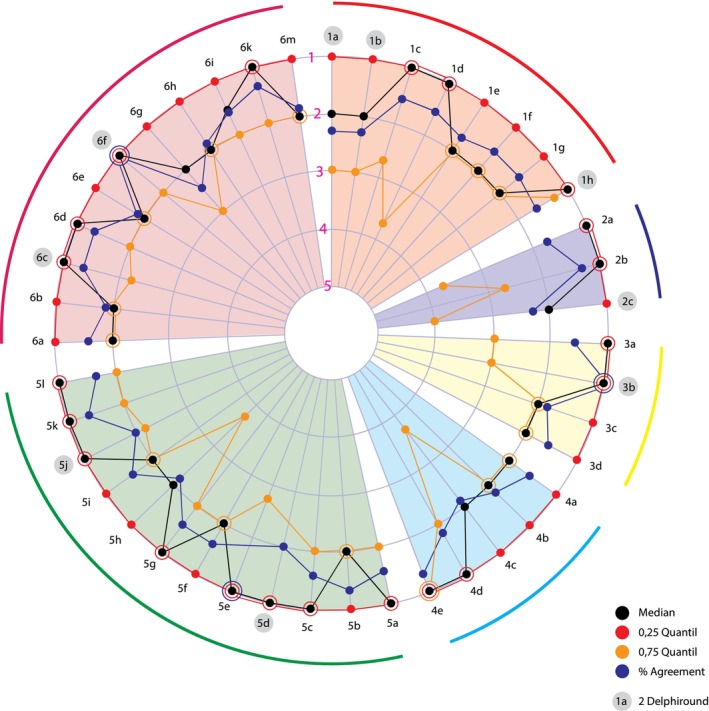
The six domains (1. Care dependency in older individuals, 2. Access and barriers to oral health, 3. Legal aspects, 4. Communication, 5. Effects of impairments, diseases, and age on oral health 6. Clinical management of (DOP)) including the voting results for the individual learning objectives (1a‐6m). The pink numbers mark the vote: 1 (on the outer circle) = maximum agreement, 5 (inside the circle) = maximum disagreement.

Of the 20 experts invited, 14 (70%) participated in the first Delphi round, held in July 2024. In the second round, conducted in August 2024, 12 experts (60%) participated, all of whom were a subset of those who took part in the first round. A consensus was reached for all LOs in the second round, with consensus defined as at least ‘moderate agreement’. A total of six competence areas and 43 LOs were consented (Table 1, Figure 2).

**TABLE 1:** Six domains with the learning objectives and results of the Delphi survey.

Domain	Learning objectives	Delphi round	Modus	Median	0.25‐Quantil‐	0.5 ‐Quantil	0.75‐Quantil	IQR	Agreement (%)
1. Care dependency in older individuals	1a. Describe the cultural, legal, and social context of care dependency in older age.	1	1	1.5	1	1.5	2	1	80.75
		2	2	2	1	2	3	1	66.67
	1b. Explain the International Classification of Functioning, Disability and Health.	1	2	2	1	2	3	2	57.14
		2	1	2	1	2	3	2	66.67
	1c. Explain age‐related diseases, multimorbidity, frailty.	1	1	1	1	1	1.75	0.75	85.71
	1d. Foster a positive attitude towards human diversity and ageing.	1	1	1	1	2	2.75	1.75	85.71
	1e. Describe theories of ageing, the biology and physiology of ageing.	1	1	2	1	1	2	1	71.43
	1f. Explain demographic characteristics and trends in DOP.	1	2	2	1	2	2	1	85.71
	1g. List various care concepts for DOP.	1	2	2	1	2	2	1	85.71
	1h. List and explain the components of comprehensive geriatric assessments.	1	1	2	1	2	2.75	1.75	71.42
		2	1	1	1	1	1.25	0.25	83.33
2. Access and barriers to oral health	2a. Identify and list reasons for barriers to health service utilisation by DOP.	1	1	1	1	1	2.75	1.75	71.42
	2b. Describe measures to facilitate access to dental care for DOP.	1	1	1	1	1	1.75	0.75	92.86
	2c. Describe the terms ‘ageism’ and ‘ableism’ and explain them with examples in the dental context.	1	1	2.5	1	2.5	3	2	50.00
		2	2	2	1	2	3	2	66.67
3. Legal aspects	3a. Describe the process of consent and informed consent in the dental care of older people with communicative, cognitive or sensory impairments.	1	1	1	1	1	2	1	85.71
	3b. Describe the relevance of patient autonomy and the role of family and caregiving staff in supported decision‐making.	1	2	2	1	2	2	1	92.86
		2	1	1	1	1	2	1	100.00
	3c. Name the billing positions related to § 22a SGB V, as well as their indications and implementation modalities.	1	2	2	1	2	2	1	78.57
	3d. Describe the relevance of existing legal guardianship in dental treatment planning.	1	1	2	1	2	2	1	85.71
4. Communication	4a. Describe appropriate communication methods for people with cognitive, sensory and/or other communication impairments.	1	1	2	1	2	2	1	85.71
	4b. Apply appropriate communication methods to people with cognitive, sensory and/or other communication impairments.	1	2	2	1	2	2	1	78.57
	4c. Communicate in a culturally sensitive and integrative language with patients, colleagues and caregiving staff.	1	2	2	1	2	2.75	1.75	71.43
	4d. Conduct consultative conversations and request consultations.	1	1	1	1	1	2	1	78.57
	4e. Instruct and motivate caregiving and lay staff in conducting oral care measures for DOP.	1	1	1	1	1	1	0	92.86
5. Effects of medical and functional condition on oral health	5a. Describe key elements of impairments, disabilities and systemic diseases that can affect oral health in DOP.	1	1	1	1	1	2	1	85.71
	5b. Recognise the need for interdisciplinary collaboration in patient assessment and list its benefits.	1	2	2	1	2	2	1	92.86
	5c. Describe common health problems, diseases and multimorbidity in seniors.	1	1	1	1	1	2	1	85.71
	5d. Describe the principles of pharmacodynamics and pharmacokinetics in older patients and explain their dental relevance.	1	1	1	1	1	2	1	85.71
		2	1	1	1	1	2.25	1.25	75.00
	5e. Describe side effects of medications and their effects on oral health.	1	1	1	1	1	1.75	0.75	100.00
	5f. Describe neurological and psychological disorders in older people and their impact on oral health competence.	1	2	2	1	2	2	1	85.7
	5g. Assess the dental treatment capacity and resilience, as well as oral hygiene ability of a DOP.	1	1	1	1	1	2	1	85.71
	5h. Quantify oral health‐related quality of life in DOP and describe appropriate tools for its assessment.	1	2	2	1	2	2.75	1.75	71.43
	5i. Describe the bidirectional effects of dental and general health.	1	1	2	1	2	2	1	85.71
	5j. Name assessments to quantify reduced oral health in people with care needs. (xx)	1	3	3	1	3	3	2	57.14
		2	1	1	1	1	2.25	1.25	75.00
	5k. Diagnose xerostomia, describe etiological factors and clinical management of the condition. (x)	2	1	1	1	1	2	1	91.67
	5l. Explain possible causes and the dental relevance of dysphagia. (x)	2	1	1	1	1	2	1	83.33
6. Clinical management of dependent older people	6a. Consider medical, social, psychological and environmental factors that should be considered in risk assessment and dental treatment planning for DOP.	1	2	2	1	2	2	1	85.71
	6b. Incorporate communicative and pharmacological approaches that can support the dental treatment of DOP with reduced cooperation ability.	1	2	2	1	2	2	1	78.57
	6c. Design and implement oral health education and oral care plans (according to §22a SGB V) for DOP and caregivers.	1	1	1	1	1	2	1	92.86
		2	1	1	1	1	1.25	0.25	91.67
	6d. Implement simple clinical treatment using appropriate measures for DOP.	1	1	1	1	1	2	1	92.86
	6e. Describe the value of teamwork in the dental treatment and care of DOP and name the individual roles in maintaining maximum oral health.	1	2	2	1	2	2	1	78.57
	6f. Take responsibility for the dental care of DOP and justify treatment decisions.	1	2	2.5	1	2.5	3	1	50.00
		2	1	1	1	1	2	1	100.00
	6g. Design and conduct training for auxiliary and caregiving staff in the basic skills of oral hygiene for DOP.	1	1	2	1	2	3	2	64.29
	6h. Describe mobile dental treatment options and concepts for outreach dental care.	1	1	2	1	2	2	1	78.57
	6i. Select individual, patient‐tailored treatment options.	1	1	1.5	1	1.5	2	1	85.71
	6k. Describe factors for a safe, calm treatment environment and identify ways to facilitate access to dental care.	1	1	1	1	1	2	1	92.86
	6m. Justify the indication for dental treatment under general anaesthesia.	1	2	2	1	2	2	1	78.57

*Note:* (x) new added in Round 2, based on the expert feedback. (XX) has been reformulated and therefore included in the second round.

Abbreviation: IQR, interquartile range.

Based on the agreed‐upon LOs and expert feedback gathered during the Delphi process, a Graduate Profile for dentists providing dental care for DOP was developed (Table 2).

**TABLE 2:** Graduate Profile for dentists providing dental care for dependent older people (DOP).

Domain	At the end of their studies, dental students in Germany will be competent to
1. Care dependency in older individuals	1a. Describe the cultural, legal and social context of the need for care in old age.
	1b. Explain the physiology and heterogeneity of ageing and their significance for oral health.
	1c. Name age‐associated diseases and explain them in the context of multimorbidity and frailty.
	1d. Analyse national demographic characteristics and trends in dependent older people (DOP).
2. Access and barriers to oral health	2a. Define the term ‘ageism’ (age discrimination), explain it using examples in a dental context and identify strategies to combat it.
	2b. Identify barriers to the use of dental services for DOP and develop solutions.
	2c. Plan and describe measures to facilitate access to dental care for DOP, including accessible practice design and mobile dental care.
3. Legal aspects	3a. Explain the process of consent and informed consent in dental care for older people with communicative, cognitive or sensory impairments.
	3b. Explain the importance of patient autonomy and consider the role of family and carers in supported decision‐making processes.
	3c. Explain financing systems and their areas of application as well as implementation modalities (e.g., billing items for DOP with statutory health insurance in Germany/§ 22a SGB V).
	3d. Describe and take into account the significance of existing statutory care in dental treatment planning.
	3e. Recognise signs of abuse or neglect in DOP, document them appropriately and take the necessary ethical and legal steps to safeguard the patient's welfare.
4. Communication	4a. Use appropriate communication methods with older people with cognitive, sensory and/or other communication impairments.
	4b. Train and motivate caregivers and lay staff in the implementation of oral hygiene measures for DOP.

4c. Demonstrate effective communication skills within an interprofessional healthcare team to collaboratively develop and implement patient‐centred dental treatment plans, ensuring comprehensive and holistic care.
5. Effects of medical and functional condition on oral health	5a. Explain the bidirectional relationship between general health, care needs and highly prevalent oral diseases in older age (severe periodontitis, root caries and dry mouth).
	5b. Consider pharmacotherapy in older patients in dental care and evaluate its impact.
	5c. Analyse and discuss the impact of oral health/oral disease on patients' quality of life and oral function, with a particular focus on the importance of preventative and therapeutic strategies.
	5d. Explain possible causes and dental relevance of dysphagia.
6. Clinical management of dependent older people	6a. Design the dental treatment plan taking into account therapeutic capability and oral hygiene skills.
	6b. Implement concepts for oral health education and oral hygiene planning (according to §22a SGB V)^a^ for DOP and caregivers.
	6c. Identify patients with support needs and consider diseases in dental treatment planning and therapy.
	6d. Describe different dental care settings (e.g., dental practice, mobile dental care, public health service) and discuss them in relation to different case studies.
	6e. Diagnose highly prevalent oral diseases in DOP (e.g., root caries, severe periodontitis and dry mouth), analyse their aetiological factors and implement effective treatment plans, including preventive and restorative approaches.

^a^ In accordance with the statutory health insurance regulations in Germany, individuals with a care level or receiving integration assistance are entitled to additional dental services, including biannual assessments of oral health, personalised oral health plans, and treatments such as the removal of calculus.

## 4 Discussion

Our work shows that the subject catalogue (CES), as the basis for the teaching content of the German dental curriculum, contains all important competencies for providing oral health care to care dependent older adults. Our developed LOs represent therefore no additional content, but show a condensate based on international recommendations [24, 31], German guidelines and expert consensus, and thus enable the development of a structured curriculum. The consensus achieved through the Delphi process highlights both the relevance and the necessity of specific LOs for providing adequate dental care to DOP. Integrating these LOs into the curriculum is more crucial than ever, as the rapidly growing older population not only lives longer but also faces an escalating burden of complex oral health challenges driven by a higher prevalence of multimorbidity, cognitive decline, and frailty [34, 35]. Additionally, due to improved prevention in earlier, healthier life stages, many patients retain their natural teeth longer, further increasing the need for specialised dental care. The high level of consensus reached is clear evidence of the need to prepare future dentists for the unique challenges posed by treating DOP. Most LOs achieved very high agreement in relevance (median 1, IQR 0, ≥ 80% scoring 1 or 2), reflecting the widespread recognition among experts of this necessity. LOs that did not reach consensus were predominantly characterised by overlapping content with existing items, limited perceived relevance for undergraduate curricula, or a level of specialisation more appropriate for postgraduate training. These items were therefore excluded after discussion within the core research team.

A potential critique of this study is that the experts involved may be biassed, given their direct involvement in the care of older patients with special dental needs. While dental professionals with no direct experience of dealing with DOP might not view these findings as critical, the existing evidence underscores the relevance of integrating such care into curricula. This integration is therefore not only necessary but imperative to ensure comprehensive dental education and improve patient care outcomes.

The alignment of the LOs from international guidelines [24, 31] with the standard LOs of the German dentistry curriculum demonstrates that the German framework (CES) encompasses the key competencies outlined in international guidelines. This represents a critical step towards standardising dental education in the field of dental care provision for care‐dependent individuals.

One of the notable contributions of this study is the development of a detailed graduate profile that aligns with the consented LOs. This profile may serve as a blueprint for educators to increase graduate dentists' competencies in dental care provision for DOP. The profile also provides a framework for assessing both skills and attitudes—two critical components in ensuring that graduates are not only capable but also willing to treat this growing and often underserved population [36].

The quartile data (1st, 2nd and 3rd) revealed that most responses were clustered closely together. The 1st quartile was consistently rated as 1, while the 3rd quartile showed some variance, with values like 2 or 3. This indicates that while most responses were concentrated around the median, there is a slight spread towards higher ratings for some items. The range of responses, as represented by the 1st and 3rd quartiles, suggests that there is limited variation in perceptions or assessments across the different criteria. This trend points towards a generally homogeneous response pattern. The third quartile often had values closer to 2 or slightly higher, indicating that a significant portion of respondents lean towards relevant evaluations of the LOs. The modus (most frequent score) and median were often the same across different items, which further confirms the homogeneity in responses.

Several key areas emerged as critical in the education of dental students regarding DOP, including communication, understanding the effects of age‐related diseases, and clinical management. The high level of agreement in these domains suggests that there is a shared understanding among experts about the competencies needed. However, as GD does not have academic chairs in Germany and there are only very few dentists who are experts in providing dental treatment for DOP and also experts in dental education, the number of participants (14 experts in the first round, 12 in the second round) can be seen as positive [19].

To achieve the LOs identified in this study, GD must be properly integrated into the entire dental curriculum. Curriculum surveys underscore these gaps: across Europe, 86.2% of schools report teaching gerodontology, yet only 64.2% provide clinical teaching and just 26.8% offer outreach experiences [17, 18].

It is widely accepted that integration is needed to make the learning more relevant and ultimately more available for use in a clinical context [37]. Case‐based learning strategies are recognised as most appropriate for such integration, but varying levels of problem orientation may be suitable in different situations [38]. Interactive elements like simulations of ageing‐related impairments [39], role‐playing with standardised patients, and training in preventive strategies for DOP.

Additionally, clinical‐practical teaching activities at external locations, such as day centres, nursing homes and geriatric hospitals, must be integrated into the curriculum. These outreach experiences provide students with valuable hands‐on learning opportunities in realistic care settings, fostering their ability to address the unique needs of DOP.

Beyond dental students, comprehensive care for DOP requires close interprofessional collaboration with nursing, geriatric medicine, social care and allied health professionals [4, 7, 24]. Oral hygiene routines, early detection of oral problems and the organisation of access to dental services are often delivered by nursing staff and caregivers rather than dentists themselves. Therefore, curricula should not only prepare dental students but also create structured interfaces with programmes in nursing and geriatric care. Interprofessional learning formats, such as joint simulation sessions, shared case discussions and outreach activities in long‐term care facilities, may strengthen communication, mutual understanding of roles and continuity of oral healthcare within the broader care network [40]. Such formats help to close existing gaps in the care pathway that cannot be addressed by dental training alone.

Furthermore, the curriculum must incorporate emerging fields relevant to GD, such as digital technologies tailored to the ageing population, like assistive devices for mobile dental care [41, 42]. These ‘new sciences’, although not traditionally linked to dentistry, are vital in preparing students for the growing challenges in older patient care.

### 4.1 Proposal for Implementation in the German Curriculum

The consensus‐based learning outcomes provide a concrete framework to support the development of teaching and assessment formats in gerodontology. Given the current heterogeneity of gerodontological training across faculties, these results offer guidance for aligning curricula with competence‐based standards and for integrating structured assessment formats such as structured oral examinations. By offering clearly defined and measurable competencies, the findings may also inform ongoing national discussions on curricular harmonisation and contribute to more comparable educational standards across dental schools in Germany.

**FIGURE 3:**
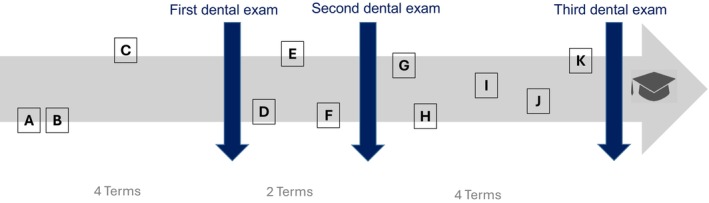
Exemplary implementation of the new learning objectives in the German dental curriculum. (A) Introduction to geriatrics and the need for care: Interactive lectures and seminars that deal with the cultural, legal and social context of the need for care could form the basis. (B) Career exploration and nursing service: Students should complete internships that take place on geriatric wards (e.g., geriatrics or care facilities). In addition, an insight into outreach dental care in care facilities could be provided as part of the career exploration programme (Berufsfelderkundung). (C) Simulation exercises: The use of ‘instant ageing’ suits would allow students to experience age‐related limitations (e.g., hearing loss, limited mobility) and promote sensitisation to the challenges of dealing with elderly patients. (D) Communication exercises: Seminars and role plays could be specifically designed to train communication with older people with cognitive, sensory or communicative impairments. Dealing with relatives and carers could also be addressed. (E) Integration of geriatric topics in phantom courses: For example, filling therapy for root caries could be practised on appropriate models. (F) Problem‐oriented learning (POL): Case vignettes that address preventive measures in multimorbid patients could help students to develop interdisciplinary approaches and recognise the importance of cooperation with nursing science and geriatrics. (G) Integrated treatment courses: In these courses, students could develop and implement treatment strategies for elderly patients and patients in need of care under supervision. Medical, psychological and social factors should be taken into account. (H) Work shadowing in care facilities: Building on the experiences from the first stage of the programme, students could visit care facilities again and develop preventative and therapeutic concepts for individual residents. These cases could be documented in writing and presented in interactive seminars. (I) QB ‘Medicine and dentistry of ageing and older people’: Offers an excellent opportunity to specifically anchor the care of elderly people and people in need of care in dental training. (J) Other interdisciplinary modules: The other eight QBs could also be used, for example, to deepen the interaction of general diseases and oral health in people with care needs and ethical issues. (K) Teamwork and training: Students could learn to instruct carers in oral hygiene and reflect on the importance of interdisciplinary cooperation in the care of elderly patients.

Recognising the challenges of integrating additional content into already crowded curricula, the LOs were carefully designed to be easily adaptable. The study was methodologically structured to ensure that the LOs align with German and European guidelines. This approach allows the outcomes to fit into the general profiles and competencies defined by global educational associations, while also meeting the requirements of professional regulatory bodies worldwide. The learning LOs provide a substantive framework for the design of the newly mandatory interdisciplinary lectures in the German dental curriculum: ‘Medicine and dentistry of ageing and older people’. It is important to note that one course, in isolation, will not provide a comprehensive curriculum. To ensure a thorough and balanced educational experience, it is advisable to integrate the learning content into the curriculum in a spiral, and to administer regular assessments (Figure 3). The content should be aligned with the core competencies of other specialties to ensure coherence and avoid redundancies.

Contents on DOP as a vulnerable, growing population group, as well as effects of functional decline on oral health should be taught in the first study phase (semesters 1–4). Early contact is desirable in order to reduce fear of contact with DOP. The professional field exploration (‘Berufsfelderkundung’), which consists of both theoretical and practical components, provides ideal opportunities to introduce the students to the topic of GD. By the second phase of the curriculum (semesters 5–6), students are introduced to general skills necessary for dental practice, primarily through phantom courses. Although these courses focus on foundational dental skills, lectures and seminars should also address the specific needs of DOP, such as root surface restorations. Communication exercises should emphasise the particular needs of DOP. Practical training should include oral hygiene for DOP. In the third phase (semesters 7–10), clinical aspects and clinical training take precedence, focusing on the application of (phantom‐based) training and the development of treatment strategies. Interdisciplinary modules (‘Querschnittsbereiche’, QBs), such as ‘Medicine and Dentistry of Ageing and older people’, ‘Pain medicine’ and ‘Oral Medicine and Systemic Aspects’, should address specific geriatric issues. The ‘medicine and dentistry of ageing and older people’ module is ideally conducted in an interprofessional block format addressing legal aspects, access and barriers to oral health and clinical management of DOP (Figures 2 and 3).

The need for structured teaching and assessment concepts is not limited to Europe. International reports from the United States, Canada, and Australia also highlight the challenges of integrating gerodontological competencies into dental curricula, underscoring the global relevance of this issue [17, 43]. At the same time, concrete educational frameworks and evaluated teaching approaches exist, including the European College of Gerodontology undergraduate curriculum guidelines [24] and simulation‐based or modular geriatric teaching programmes that demonstrated measurable competency gains [38, 39].

The continuous adaptation of the LOs from a scientific perspective must be led by the faculties. When resources are limited, prioritisation of LOs can facilitate the rational allocation of those resources. If not all of the content can be implemented, extracurricular/postgraduate training programmes are needed to ensure dental care for DOPs in the long term. It is essential to assess the need for specialised professionals and define the required competencies. Furthermore, strategies must be developed to attract dentists to teaching and consultancy roles in gerodontology. Establishing a specialisation pathway could provide clear career prospects in academia, public service, and private practice.

## 5 Conclusion

In concordance with European and German guidelines for dental education this study provides a comprehensive educational framework addressing German dental graduates' competences necessary to provide dental care to DOP. Future research should focus on the implementation and effectiveness of these LOs in practice, as well as the long‐term impact on patient outcomes and student preparedness. This study highlights the critical role of education in reducing health inequalities and equipping dental professionals with the confidence and skills to care for all members of society. However, further research is needed to explore this topic in greater depth.

## Funding

The authors have nothing to report.

## Conflicts of Interest

The authors declare no conflicts of interest.

## Supporting information


**Table S1:** eje70094‐sup‐0001‐TableS1‐S14.docx.

## Data Availability

All data supporting the findings of this study are provided in the Supporting Information.
