# Supplementary material for: Competences for Providing Oral Health Care to Care‐Dependent Older Adults—Defining Learning Objectives for the German Undergraduate Dental Curriculum Through a Delphi Study
Source: Eur J Dent Educ. 2026 Jan 16;30(3):1369–81. doi: 10.1111/eje.70094 (PMC13383296; doi:10.1111/eje.70094)
Supplement: Supplementary file 1 — Table S1: eje70094‐sup‐0001‐TableS1‐S14.docx. [file EJE-30-1369-s001.docx]

| Learning contents from the German **Catalogue of Examination Subjects (**impp-Gegenstandskatalog), the European College of Gerodontology: Undergraduate curriculum guidelines and the Guidance for the core content of a Curriculum in Special Care Dentistry at the undergraduate level were mapped to the six major domains. On the basis of the overlapping content of this three guidelines new learning objectives were developed, which were agreed as part of the Delphi process and streamlined to emphasize core competencies. | | | | |
| --- | --- | --- | --- | --- |
| **Catalogue of Examination Subjects**  For the Written Part of the Third Section of the Dental Licensing Examination First Edition, December 2023 | **curriculum in**  **Gerodontology** (Kossioni et al. 2009)  1-29 = The dentist must be competent at..  30-45 = The dentist must have knowledge of..  46-50 = The dentist must be familiar with.. | **curriculum in Special Care**  **Dentistry** (Dougall et al. 2014)  Students who have successfully completed this component will be able to….  a = Knowledge (cognitive)  b = Skills (psychomotor)  c = Behaviours (affective) | **Consented Learning Objectives after the Second Delphi Round**  These are learning objectives that reached consensus on their relevance during the second Delphi round.  At the end of their studies, students will be able to… | **Graduate profile**  At the end of their studies, students will be able to… |
| **1. care dependency in older individuals** | | | | |
| **VII.1b.2.8 Etiopathology and Pathophysiology of Age-Related Diseases**  **VII.1b.2.8.1** Etiopathology, pathophysiology, and histopathology of age-related diseases and the conditions mentioned in Chapter VI  **VII.1b.2.8.2** Explanation of age-related diseases, disorders, changes, and symptoms, as well as the derivation of diagnostics and therapy  **VII.1b.2.8.3** Pathogenetic mechanisms of aging  **VII.2.1.5 Methodological Foundations of Age-Related Examinations**  **VII.2.1.5.1** Age-specific general and disease-specific physical examination in infants, toddlers, school-age children, and adolescents (VIII.7.2.6.2)  **VII.2.1.5.2** Specialized geriatric examinations and test procedures  **VII.3.1.11 Concepts of Acute Geriatric Rehabilitation**  **VII.3.1.11.1** Concepts of inpatient acute geriatric rehabilitation  **VII.3.1.11.2** Concepts of outpatient acute geriatric rehabilitation  **VIII.2.5.3.1 Effects on Health, Disease, and Disability**  **VIII.4.2 Health Psychology – Health, Disease, and Health Behavior of Individuals and Society**  **VIII.4.2.6** Coping with and managing chronic diseases  **VIII.4.2.6.1** Epidemiology of chronic diseases  **VIII.4.2.6.2** Heterogeneity and challenges of chronic diseases  **VIII.4.5.5.5 Diversity and Modifiability of Health and Disease in Aging**  **VIII.4.5.6 Participation, Inclusion, and Health**  **VIII.4.5.6.1** Definitions of participation, inclusion, and health  **VIII.4.5.7 Disability and Health**  **VIII.4.5.7.1** Disability – Definition and terminology  **VIII.4.5.7.2** Prevalence of disability  **VIII.4.5.7.3** Causes of disability  **VIII.4.5.7.4** Disability, morbidity, and mortality  **VIII.4.5.7.5** Challenges in healthcare provision  **VIII.4.5.7.6** Holistic treatment of people with intellectual disabilities  **VIII.4.5.7.7** Therapeutic relevance in disability  **VIII.6.2.5.3 Explanation of the Term "Vulnerability," Identification of Vulnerable Patient Groups, and Needs-Based Approaches (see also 4.7)**  **VIII.6.2.7.6 Essential Ethical, Legal, Social, Cultural, and Historical Aspects Related to the Treatment of All Patients with Special Needs**  **VIII.6.5.4 Social Long-Term Care Insurance (SGB XI)**  **VIII.6.5.4.1** Definition of care dependency and care levels (21.1.1.2)  **VIII.6.5.4.2** Caregiver-patient relationship (21.1.1.2)  **VIII.6.5.4.3** Medical care, basic care, and household assistance under SGB V and SGB XI  **VIII.6.5.4.4** Home care and assisted living groups (9.3.1.1) | The principal demographic characteristics and trends in the aged population. (30)  Physiological and pathological age-related changes. (31)  Major neurological and psychological disturbances in the aged (memory impairment, pain  perception, changes in anxiety, self-esteem and disorientation). (36)  Appropriate laboratory diagnostic tests for the most common geriatric diseases and the  interpretation of the results. (38)  The principles of pharmaco-dynamics and pharmaco-kinetics in the elderly patient.(40)  Drug interactions and relevance of polypharmacy. (41)  Concepts of death and dying. (50)  The use of geriatric assessment scales (dementia, depression, nutrition). (49)  The principles of management of geriatric medical conditions. (48)  Theories of ageing. (46) | Describe the cultural, legal and social context of people with disability and other marginalised groups. (1a)  Discuss epidemiology, terminology, concepts & classifications of human function, disability (1b)  Demonstrate positive attitudes in relation to human difference and diversity. (1c) | 1a. Describe the cultural, legal, and social context of care dependency in older age.  1b. Explain the International Classification of Functioning, Disability, and Health.  1c. Explain age-related diseases, multimorbidity and frailty.  1d. Foster a positive attitude towards human diversity and aging.  1e. Describe theories of and the physiology of aging.  1f. Explain demographic characteristics and trends in DOP.    1g. List various care concepts for DOP.  1h. List and explain the components of comprehensive geriatric assessments. | 1a. describe the cultural, legal, and social context of care dependency in older age.  1b. describe the physiology and heterogeneity of aging.  1c. explain age-related diseases, multimorbidity and frailty.  1d. explain demographic characteristics and trends in DOP. |
| **2. Access and barriers to oral health** | | | | |
| **VIII.2.5.3 Societal Stigmatization Processes**  **VII.2.1.5.4 Medical Care Context**  **Application examples:** Ethical, social, cultural, legal, and historically relevant aspects  **VII.3.1.6.3 Individual Needs and Requirements of People with Disabilities, Care Needs, and Chronically Ill Patients and Their Support Networks**  **VII.3.7.3.4 Consideration of Psychological Abnormalities and Somatic Causes That Influence Patients' Utilization of Dental-Therapeutic or Preventive Measures**  **VIII.2.2.8.3 Health Policy and Economic Influences**  **VIII.3 Interprofessional Competencies**  **VIII.3.2.1.4** Aligning actions in interprofessional healthcare with individual patient interests and their environment  **VIII.3.2.2.6** Ensuring continuity of patient care on a case-by-case basis  **VIII.3.2.3** Collaboration with other healthcare professions  **VIII.3.2.3.1** Role as a team member in optimizing health promotion, prevention, cure, rehabilitation, and palliative care  **VIII.3.2.3.2** Inclusion of representatives from different healthcare professions across various institutions in needs assessment, planning, implementation (including problem-solving) of healthcare processes intra- and intersectorally  **VIII.3.2.3.10** Identifying the health literacy of patients and their support networks  **VIII.4.2.3.1 Health-Related Protective and Risk Factors for Health and Healthcare Utilization Behavior of Individuals and Population Groups, Their Relative Significance, and the Available Evidence**  **VIII.4.2.3.2 External Influences on Health Behavior – Everyday Environment and Social Structures (9.1.2.3) (9.2.2.3) (9.2.1.3)**  **VIII.4.2.3.4 Various Influencing Factors and Parameters (Genetic, Behavioral, Environmental) and Their Impact on Oral Health and Overall Health Status (9.1.1.3) (9.2.1.2) (9.2.1.3) (19.1.3.1)**  **VIII.4.2.3.5 Intersectionality**  **VIII.4.2.3.9 Individual Barriers and Incentives for Measures to Improve Oral Health and Related Overall Health Status (9.1.2.3) (9.1.1.4) (9.2.2.3)**  **VIII.4.4.1.1 Empirical Findings on Social Differences in Morbidity and Mortality**  **VIII.4.4.1.2 Life-Stage and Life-Course Considerations (Childhood, Adolescence, Young Adulthood, Adulthood, Old Age) (9.2)**  **VIII.4.4.2 Dimensions of Health Inequality (9.2.1)** | Recognising the presence of the major systemic diseases in old age and how they affect the  delivery of oral care (12)  Suggesting strategies to overcome barriers to dental care for the elderly patients. (20)  The organisation of general and oral health care for the elderly in the community and in the  hospitals and the organisation of domiciliary care (47) | Identify the social determinants of health in relation  to health inequalities in people with disability  and other marginalised groups. (2a)  Recognise barriers and facilitators to oral health  for people with disability and other  marginalised groups. (2b)  Use social and environmental facilitators to  oral health and oral health promotion  within service structure.(2c) | 2a. Identify and list reasons for barriers to health service utilization by DOP.  2b. Describe measures to facilitate access to dental care for DOP.  2c. Describe the terms "ageism" and "ableism" and explain them with examples in the dental context. | 2a. describe and combat the term "ageism" and explain it with examples in the dental context.  2b. identify for barriers to dental health service utilization for DOP.  2c. describe and propose measures to facilitate access to dental care for DOP, including the implementation of barrier-free practice designs and the provision of mobile dental services. |
| **3. Legal aspects** | | | | |
| ****VII.3.1.9.2 Legal Framework for Treatment Planning (22.1.1.5)********VIII.4.5.5.6 Guardianship Law********VIII.4.5.5.7 Advance Directives and Living Wills********VIII.6.2.5.4 Consideration of the Practice of Legal Representation of Patients and Its Normative Implications (18.3.1.5)********VII.5.1.4.2 Neglect, Abuse, and Violence – Risk Factors, Signs, and Preventive Measures********VII.5.5.2.1 Various Forms of Violence and Neglect, Their Consequences, and Appropriate Interventions**** | Procedures in managing patients with reduced ability to consent (44) | Outline the appropriate consent process when providing  care for people with communication, cognitive  or sensory impairments. (3a)  Obtain valid consent or assent for oral health  procedures appropriately. (3b)  Demonstrate respect for patient  autonomy and the role of the family  and caregivers in supported decision making.(3c) | 3a. Describe the process of consent and informed consent in the dental care of older people with communicative, cognitive, or sensory impairments.  3b. Describe the relevance of patient autonomy and the role of family and caregiving staff in supported decision-making.  3c. Name the billing positions related to § 22a SGB V, as well as their indications and implementation modalities.  3d. Describe the relevance of existing legal guardianship in dental treatment planning. | 3a. describe the process of consent and informed consent in the dental care of older people with communicative, cognitive, or sensory impairments.  3b. describe the relevance of patient autonomy and the role of family and caregiving staff in supported decision-making.  3c. explain funding schemes as well as their indications and implementation modalities. (billing items for legally insured DOPs in Germany/§ 22a SGB V)  3d. describe the relevance of existing legal guardianship in dental treatment planning.  3e. identify signs of abuse or neglect in DOP, document them appropriately, and take necessary ethical and legal actions to ensure patient safety and well-being. |
| **4. Communication** | | | | |
| **VII.3.4.3.8 Integration of Dental Prostheses (23d.3.2.13) Application examples:** Instruction of staff in cases of patients requiring care  **VII.3.7.3 Principles and Fundamentals of Conversational Therapy and Their Application in Relation to the Situation, Patients, and Their Impact on Treatment Planning, Therapy, and Prognosis**  **VII.4.3.1.2 Collaboration with Other Medical Specialists, Including Conducting Consultative Discussions and Efficiently Requesting Consultations**  **VIII.2 Dental Communication Skills**  **VIII.2.3.2.2 Challenging Situations in the Dentist-Patient Relationship (7.3, 7.3.1.2)**  **VIII.2.3.2.3 Supportive and Palliative Interventions for Seriously Ill Patients**  **VIII.2.4.3 Conversations with Relatives and Their Influencing Factors**  **VIII.2.4.3.1 Patient Autonomy, Confidentiality, and Privacy**  **VIII.2.5.1 Age- and Gender-Appropriate Communication (7.5)**  **VIII.2.5.1.1 Age-, Development-, Disability-, and Gender-Related Variables Influencing Communication (7.5.1.1)**  **VIII.2.5.2 Sociocultural Diversity**  **VIII.2.5.2.1 Sociocultural Diversity**  **VIII.2.5.2.2 Consequences of Language Barriers**  **VIII.2.5.4 Dealing with Perception and Communication Disorders**  **VIII.2.5.4.1 Perception and Communication Disorders**  **VIII.3.2.2.4 Necessity of Consultation with Treating General Practitioners or Specialists**  **VIII.3.3.1.3 Recognizing Emotions in Interactions with Colleagues and Reflecting on Processes of Transference and Countertransference**  **VIII.3.3.1.4 Open, Respectful, and Appreciative Attitude and Interaction as the Basis for Team Relationships**  **VIII.3.4.1.7 Establishing Appropriate Relationships with Patients and Their Relatives, Colleagues, Nurses, and Other Professional Groups in Accordance with the Professional Role**  **VIII.3.4.2 Roles of Representatives of Different Health Professions**  **VIII.3.4.2.1 Roles, Competencies, and Responsibilities of Representatives of Different Professions in Various Institutions in Health Promotion, Prevention, Cure, Rehabilitation, and Palliative Care**  **VIII.3.4.1.6 Own Responsibilities and Tasks in Collaboration with Other Dentists and Physicians (from Clinics, Private Practices, Assistance, and Consultative Services)**  **VIII.3.2.3.9 Aspects of Interprofessional Healthcare and Treatment for Children and Adults with Intellectual or Multiple Disabilities** | Displaying an appropriate and ethical caring behaviour towards older patients (1)  Communicating effectively with the aged dental patient taking into account the physical,  psychological and mental status of the patient (5)  Communicating effectively and sharing information with all members of the health care team  (physicians, nurses, dental assistants, hygienists etc.) and the carers (7)  Performing a written referral to clarify the patient’s general condition (11)  Providing oral education and oral hygiene instructions to the older patient and particularly to  patients with diminished manual dexterity (17)  Training auxiliaries and carers in basic skills of oral hygiene for the frail and dependent aged.(18)  Training auxiliaries and carers in the perception of pain and oral impairment for dental referral. (19)  The oral health-care management of people with cognitive impairment. (43) | Describe appropriate methods of communication for  people with cognitive, sensory and/or other  communication impairments. (4a)  Use appropriate methods of communication  for people with cognitive, sensory and/or  other communication impairments. (4b)  Demonstrate culturally sensitive and  inclusive language with patients,  colleagues and care givers. (4c) | 4a. Describe appropriate communication methods for older people with cognitive, sensory, and/or other communication impairments.  4b. Apply appropriate communication methods to older people with cognitive, sensory, and/or other communication impairments.  4c. Communicate in a culturally sensitive and integrative language with patients, colleagues, and caregiving staff.  4d. Conduct consultative conversations and request consultations.  4e. Instruct and motivate caregiving and lay staff in conducting oral care measures for DOP | 4a. apply appropriate communication methods to older people with cognitive, sensory, and/or other communication impairments.  4b. instruct and motivate caregiving and lay staff in conducting oral care measures for DOP  4c. demonstrate effective communication skills within an interprofessional healthcare team to collaboratively develop and implement patient-centered dental care plans, ensuring comprehensive and holistic treatment. |
| **5. Effects of medical and functional condition, on oral health** | | | | |
| **VII.4.9.3 Impact of Multimorbidity on Dental Treatment**  **VII.4.9.4 Effects of Systemic Diseases and Musculoskeletal Disorders on Dental Treatment**  **VII.4.9.6 Effects of Neurological Disorders on Dental Treatment**  **VII.4.9.8 Impact of Limited Cognitive and/or Motor Abilities on Dental Treatment**  **VII.4.9.7 Effects of Mental and Behavioral Disorders on Dental Treatment**  **VII.4.9.10 Effects of Sensory System Disorders on Dental Treatment**  **VII.1b.2.8.4 Age-Related Changes in the Salivary Glands and Their Effects on Dental Treatment (23g.10.1.11)**  **VII.1b.2.8.5 Age-Related Changes in the Oral Mucosa and Perioral Region and Their Consideration in Treatment**  **VII.3.3.7 Therapy of Salivary Gland Diseases and Their Impact on Treatment Planning and Prognosis**  **VII.3.3.7.1** Substances that Locally Stimulate Salivary Secretion (14.3.1.5) **Application examples:** Chewing gum, sour candies, locally: citric acid  **VII.3.3.7.2** Substances that Inhibit Salivary Secretion (14.4.1.2)**Application examples:** Systemically: atropine, alpha-sympathomimetics, alpha-sympatholytics; Locally: botulinum toxin  **VII.3.3.7.3** Management of the Consequences of Dry Mouth and Preventive Measures (25.2.1.5) **Application examples:** Increased caries risk; intensified caries prevention including dietary and pharmacological approaches, saliva substitutes, age-/medication-induced xerostomia  **VII.3.5 Therapy for Musculoskeletal and Soft Tissue Disorders and Their Impact on Treatment Planning and Prognosis**  **VII.3.6.2.3 Typical Side Effects of Medications or Other Therapeutic Measures on the Oral Mucosa (23f.6.1.1)Application examples:** Methotrexate, cyclosporine**. Effects:** Xerostomia, hyperplasia, bisphosphonate therapy, immunosuppressive therapy, chemotherapy, radiotherapy  **VII.3.6.2.4 Therapeutics for the Treatment of Dry Mouth (23f.6.1.2)**  **VIII.4.2.3.3 Influence of Age- and Gender-Specific Predictors and Risk Factors (19.1.3.5)**  **VIII.4.2.6.3 Consequences of Chronic Diseases**  **VIII.6.2.7.10 Dental Responsibilities for Patients in the Final Stage of Life and Consideration of Their Needs and Those of Their Relatives** | Age-related changes in special senses (sight, hearing, smell and taste). (32)  Assessing patients’ comprehension and competency.(6)  Recognising the presence of the major systemic diseases in old age and how they affect the  delivery of oral care (12)  Identifying the age-related changes in the oral structures. (14)  Identifying nutritional deficiencies, performing dietary analysis and providing nutritional advice (16)  Diagnosing xerostomia, its aetiological factors and managing the condition (23)  Common medical conditions in the elderly population. (33)  Relevance and incidence of co-morbidity. (34)  The effect of loss of status, health, family, employment, income and companions on the behaviour  and attitude of the aged. (37)  The oral manifestations of systemic diseases. (39)  Side effects of drugs and their impact on oral health. (42) | Describe common impairments, disabilities and  systemic conditions in relation to their impact  on oral health and oral function. (5a)  Identify the key elements of impairments,  disabilities and systemic conditions that may  impact on oral health or oral function for  individual patient (5b)  Consider the need for and benefits of  inter-professional liaison in patient  assessment. (5c) | 5a. Describe key elements of impairments, disabilities, and systemic diseases that can affect oral health in DOP.  5b. Recognize the need for interdisciplinary collaboration in patient assessment and list its benefits.  5c. Describe common health problems, diseases, and multimorbidity in seniors.  5d. Describe the principles of pharmacodynamics and pharmacokinetics in older patients and explain their dental relevance.  5e. Describe side effects of medications and their effects on oral health.  5f. Describe neurological and psychological disorders in older people and their impact on oral health competence.    5g. Assess the dental treatment capacity and resilience, as well as oral hygiene ability of a DOP.  5h. Quantify oral health-related quality of life in DOP and describe appropriate tools for its assessment.  5i. Describe the association between oral and general health.  5j. Describe assessments to quantify reduced oral health in people with care needs.  5k. Diagnose xerostomia, describe etiological factors and clinical management of the condition.  5l. Explain possible causes and the dental relevance of dysphagia. | 5a. Explain the bidirectional relationship between general health, care needs and highly prevalent oral diseases in the elderly (severe periodontitis, root caries and xerostomia).  5b. concider pharmacotherapy in older patients dental care.  5c. analyze and discuss the impact of oral health diseases on a patient's quality of life and function, emphasizing the importance of preventive and therapeutic strategies in improving overall well-being.  5d. explain possible causes and the dental relevance of dysphagia. |
| **6. Clinical management of dependent older people** | | | | |
| VII.4.9.1.1 Management of Treatment for Patient Groups with Special Needs, Including Prevention and Therapy Concepts, and Self-Protection **VII.3.1.10.1 Treatment Pathways Considering Special Patient Groups**  **VII.4.9.4.1 Classification of Diseases – Relationship Between Dental Symptoms, Findings, and Dental Measures (20.1.13.1)**  **VII.4.9.4.2 Prevention and Synoptic, Interdisciplinary Oral Rehabilitation in General Medical Conditions (20.1.13.2)**  **VII.4.9.8.1 Feasible and Effective Preventive Measures, Prognostically Relevant Therapy Measures, and Indications for Sedation/General Anesthesia**  **VII.3.1.5 Palliative Therapy**  **VII.3.1.5.1 Principles of Palliative Medicine and Palliative Therapy Forms. Application examples:** Definition, examples in maxillofacial surgery and geriatric dentistry (end of life care)  **VII.3.1.8.1 Therapy Planning for Relevant Medical Conditions or States Affecting the Etiology, Pathogenesis, and Therapy of Oral Diseases**  **VII.3.1.8.2 Effects of Dental Therapeutic Measures on General Medical Treatment Procedures and Medications and Their Consideration in Treatment**  **III.3.2.1.8 Interdisciplinary and Interprofessional Collaboration for Optimal Patient Treatment**  **VIII.3.2.2 Collaboration with Various Dental and Medical Disciplines**  **VIII.3.2.2.1 Proper, Appreciative, and Efficient Collaboration with Various Dental and Medical Disciplines**  **VIII.3.2.2.2 Importance of Collaboration Between Dentists, General Practitioners, and Specialists (21.1.1.1)**  **VIII.3.2.3.4 Development of Individually Relevant Therapy and Rehabilitation Goals (Interprofessional and with Patients, Relatives, and Legal Representatives if Needed)**  **VIII.3.2.3.5 Participation-Oriented Approach, Including Multidisciplinary and Interprofessional Problem Identification and Work Strategies**  **VIII.3.2.3.6 Development of a Participation Management Plan (Interprofessional and with Patients, Relatives, and Legal Representatives if Needed)**  **VIII.3.2.3.8 Adjusting the Composition of Healthcare Professionals Involved in Health Promotion, Prevention, Cure, Rehabilitation, and Palliative Care According to Developmental, Age, and Gender-Specific Differences**  **VIII.4.5.1 Prevention, (Oral) Health Promotion, and the Relationship Between Prevention and Health Promotion at the Individual and Population Level**  **VIII.4.5.1.7 (Oral) Health Promotion in Cooperation with Key Institutions and Organizations in Healthcare**  **VIII.4.5.2.4 Influence of Patient Cooperation, Including Relatives and Caregivers, on Oral Health (21.1.1.2)**  **VIII.4.5.3 Target Group-Specific Prevention and Health Promotion**  **VIII.4.5.3.1 High-Risk Strategy vs. Population-Based Strategy – Advantages and Disadvantages**  **VIII.4.5.5 Elderly People and Seniors – Focus Areas and Empirical Studies (9.2.1)**  **VIII.4.5.5.1 Preventable Risks in Old Age**  **VIII.4.5.5.2 Aspects of Maintaining Independence, Social Connections, and Quality of Life**  **VII.5.1.4 Target Group-Specific Measures and Prevention for Older People and Seniors**  **VII.5.1.4.1 Preventable Risks in Old Age and Specific Preventive Measures** | Identifying the chief complaint and the needs and demands of the older patient (2)  Obtaining a thorough general, medical, dental and social history (3)  Performing an intra- and extra-oral examination (4)  Taking and assessing radiographs (head and neck) in the aged patients (8)  Recognising oral mucosal disorders and referring accordingly (9)  Recognising signs of elder abuse and neglect and describing the methods of reporting it to the  appropriate authorities (10)  Taking the patients’ vital signs (13)  Assessing oral health related quality of life in elderly patients. (15)  Selecting individualised patient-centred treatment options (21)  Preventing and managing dental and medical emergencies in clinical dental practice (22)  Completing a wide range of dental procedures (e.g., simple extractions, management of root  caries, secondary carries, tooth wear, periodontal treatment, endodontic therapy, fixed and  removable prostheses and management of dry mouth which are common in the elderly patients).(24)  Recognising and managing the special difficulties in removable prostheses in the elderly. (25)  Managing denture-related conditions.(26)  Providing oral health care in a multidisciplinary context. (27)  Managing aged patients with compromised general health and various levels of dependency and  knowing when to refer.(28)  Providing adequate treatment in patients’ homes and long term care settings using appropriate  dental equipment. (29)  The organisation of a safe and friendly treatment environment for the older patient for easy access  to dental care. (45) | Describe the factors (medical, social, psychological  and environmental) that impact on risk assessment  and treatment planning for individual patients  requiring special care. (6a)  Discuss behavioural and pharmacological approaches  that facilitate dental treatment for individual patients  requiring special care dentistry (according to local  guidelines and protocols) (6.1 a)  Design oral health education for individual patients and their caregivers. (6b)  Provide simple clinical treatment using  appropriate facilitation techniques for patients requiring special care, likely to present to a primary care service.(6.1 b)  Recognise the value of teamwork in the  management for patients requiring  special care. (6c)  Take responsibility for  referring or arranging care for patients  with more complex needs. (6.1 c) | 6a. Include medical, social, psychological, and environmental factors that should be considered in risk assessment and dental treatment planning for DOP.  6b. Incorporate communicative and pharmacological approaches that can support the dental treatment of DOP with reduced cooperation ability.  6c. Design and implement oral health education and oral care plans (according to §22a SGB V) for DOP and caregivers.  6d. Implement simple clinical treatment using appropriate measures for DOP.  6e. evaluate the value of teamwork in the dental treatment and care of DOP and identify the individual roles in maintaining maximum oral health.  6f. Take responsibility for the dental care of DOP and justify treatment decisions.  6g. Design and conduct training for auxiliary and caregiving staff in the basic skills of oral hygiene for DOP.    6h. Describe mobile dental treatment options and concepts for outreach dental care.  6i. Select individual, patient-tailored treatment options.  6k. Describe factors for a safe, calm treatment environment and identify ways to facilitate access to dental care.  6m. Justify the indication for dental treatment under general anesthesia. | 6a. Assess the dental treatment capacity (Care dependency status) and resilience, as well as oral hygiene ability of DOP  6b. Design and implement oral health education and oral care plans (according to §22a SGB V) for DOP and caregivers.  6c. provide dental care for patients with Pre-Dependency and Low-Dependency, considering chronic systemic conditions and their potential or current impact on oral health.  6d.evaluate and discuss appropriate treatment settings (e.g., private practise, hospital) based on different dependency levels  6e. diagnose highly prevalent oral diseases in DOP (e.g. root caries, severe periodontitis and xerostomia), analyse their etiological factors and implement effective treatment plans, including preventive and restorative approaches. |
